# Quantification of left ventricular remodeling in response to isolated aortic or mitral regurgitation

**DOI:** 10.1186/1532-429X-12-32

**Published:** 2010-05-24

**Authors:** Seth Uretsky, Azhar Supariwala, Puspalatha Nidadovolu, Surinder S Khokhar, Cindy Comeau, Oleg Shubayev, Francesca Campanile, Steven D Wolff

**Affiliations:** 1 Division of Cardiology, Department of Medicine, St. Luke's-Roosevelt Hospital Center, Columbia University College of Physicians and Surgeons, 1111 Amsterdam Avenue, New York, NY 10025, USA; 2 Advanced Cardiovascular Imaging, New York, NY, USA

## Abstract

**Background:**

The treatment of patients with aortic regurgitation (AR) or mitral regurgitation (MR) relies on the accurate assessment of the severity of the regurgitation as well as its effect on left ventricular (LV) size and function. Cardiovascular Magnetic Resonance (CMR) is an excellent tool for quantifying regurgitant volumes as well as LV size and function. The 2008 AHA/ACC management guidelines for the therapy of patients with AR or MR only describe LV size in terms of linear dimensions (i.e. end-diastolic and end-systolic dimension). LV volumes that correspond to these linear dimensions have not been published in the peer-reviewed literature. The purpose of this study is to determine the effect of regurgitant volume on LV volumes and chamber dimensions in patients with isolated AR or MR and preserved LV function.

**Methods:**

Regurgitant volume, LV volume, mass, linear dimensions, and ejection fraction, were determined in 34 consecutive patients with isolated AR and 23 consecutive patients with MR and no other known cardiac disease.

**Results:**

There is a strong, linear relationship between regurgitant volume and LV end-diastolic volume index (aortic regurgitation r^2 ^= 0.8, mitral regurgitation r^2 ^= 0.8). Bland-Altman analysis of regurgitant volume shows little interobserver variation (AR: 0.6 ± 4 ml; MR 4 ± 6 ml). The correlation is much poorer between regurgitant volume and commonly used clinical linear measures such as end-systolic dimension (mitral regurgitation r^2 ^= 0.3, aortic regurgitation r^2 ^= 0.5). For a given regurgitant volume, AR causes greater LV enlargement and hypertrophy than MR.

**Conclusion:**

CMR is an accurate and robust technique for quantifying regurgitant volume in patients with AR or MR. Ventricular volumes show a stronger correlation with regurgitant volume than linear dimensions, suggesting LV volumes better reflect ventricular remodeling in patients with isolated mitral or aortic regurgitation. Ventricular volumes that correspond to published recommended linear dimensions are determined to guide the timing of surgical intervention.

## Introduction

The timing of surgical intervention for patients with mitral or aortic regurgitation often depends on the accurate assessment of the severity of the valvular insufficiency and its effect on left ventricular (LV) size [1,2]. Two-dimensional transthoracic and transesophageal echocardiography are most commonly used to assess mitral and aortic regurgitation. Published guidelines recommend the use of LV ejection fraction as well as linear measurements such as LV end-diastolic dimension (EDD) and LV end-systolic dimension (ESD) to determine the timing of surgical interventions [2]. Cardiovascular magnetic resonance (CMR) is an accurate method for quantifying the severity of mitral and aortic regurgitation [3-7]. CMR is more accurate and reproducible than two-dimensional echocardiography in the three-dimensional volumetric evaluation of LV size and function [8-14]. An important limitation of the current guidelines is that no volumetric parameters are provided for determining the timing of surgery and we currently rely on linear measurements as a surrogate for describing LV volumetric changes. The purpose of this study is to characterize the physiological relationship between regurgitant volume and LV volume with CMR in patients with mitral and aortic regurgitation.

## Methods

### Patient characteristics

This study was a retrospective analysis of patients referred for a clinical CMR evaluation between 2006 and 2009. This study was approved by the Institutional Review Board of St. Luke's and Roosevelt Hospitals. Consecutive patients were included provided 1) they had sufficient mitral or aortic regurgitation to warrant quantification of their regurgitant volume in their clinical report and 2) phase contrast images of pulmonary artery and aortic flow were acquired with a corresponding phantom image for correcting baseline flow offsets [15]. Patients whose CMR studies showed other significant cardiac abnormalities were excluded. Exclusion criteria included: more than mildly abnormal ventricular function (LV EF < 50% or right ventricular EF < 40%), regional ventricular wall motion abnormalities, more than right ventricular (RV) enlargement (RV end-diastolic volume index >115 ml/m^2^), myocardial ischemia or scar, left ventricular hypertrophy, intracardiac shunt, more than mild tricuspid or pulmonic regurgitation, and patients with an irregular cardiac rhythm. Additionally, patients were excluded from the mitral regurgitation (MR) group if they had more than minimal (>10 ml) aortic regurgitation (AR) and from the AR group if they had more than minimal (>10 ml) MR. The analysis comprised 57 patients who underwent CMR evaluation, and included 34 patients (62 ± 17 years, male 88%) with AR and 23 patients (55 ± 15 years, male 65%) with MR.

### CMR image acquisition

Patients were imaged with a 1.5-Tesla scanner using an 8-element, phased-array coil (GE Signa, EXCITE, GE Medical Systems, Milwaukee, Wisconsin, USA). Images were acquired with ECG gating and breath holding. Short and long axis cine images were acquired using a steady-state free precession pulse sequence (FIESTA) with the following parameters: TR/TE 3.3 ms/1.4 ms, 20 views per segment, FOV 35 × 35 cm, acquisition matrix 192 × 160, slice thickness 8 mm, slice gap 0 mm, flip angle 45 degrees, receive bandwidth 125 kHz. Phase contrast images were acquired perpendicular to the proximal pulmonary artery and perpendicular to the proximal aorta to quantify flow in these vessels using the following parameters: TR/TE 7.5 ms/2.9 ms, 6 views per segment, Venc 250 cm/s, FOV 35 × 35 cm, acquisition matrix 256 × 128, slice thickness 4 mm, flip angle 20 degrees, receive bandwidth 31.3 kHz. After the clinical scan was completed, additional phase contrast images were acquired of a stationary bottle of water (phantom) for baseline flow correction [15].

### CMR image analysis

Images were reviewed and analyzed using ReportCard 4.0 software. LV volumes and mass were determined using the semiautomated LV segmentation algorithm which excludes papillary muscles and trabeculations from the LV cavity and which uses a long axis image to define the position of the LV base. RV volumes were determined by manual segmentation of the short axis images. Aortic and pulmonary artery flow values were determined using the resident semiautomated algorithm. Correction for baseline offsets was performed using a phantom phase contrast image as described previously [15]. In some patients two or three flow acquisitions were made. In these cases, the flow values were averaged. Aortic regurgitant volume was determined by integrating blood flow throughout diastole. Aortic and mitral regurgitant volume were defined as per the AHA/ACC treatment guidelines: mild < 30 ml, moderate 30-59 ml, and severe ≥60 ml. Mitral regurgitant volume was determined as the difference between the LV stroke volume (as determined by endocardial segmentation) and total pulmonary artery flow. Left atrial (LA) volumes were calculated using the area-length method (LV volume = 0.85 *Area^2^/length) by averaging values from the four and two chamber views. To determine the reproducibility of AR and MR regurgitant volume, a second blinded analysis was made according to the same method.

### Statistical analysis

All analyses were carried out using a standard statistical package (SPSS Version 16.0, SPSS Inc., Chicago, Illinois). Continuous variables were reported as mean ± SD and categorical variables were reported as percentages. Correlation between regurgitant volumes and left ventricular measures and left atrial volumes were calculated using Pearson's correlation coefficient. Interobserver variability was measured using Bland-Altman analysis and Intraclass Correlation Coefficient. P values were considered significant at < 0.05.

## Results

### Aortic regurgitation

**Figure 1:**
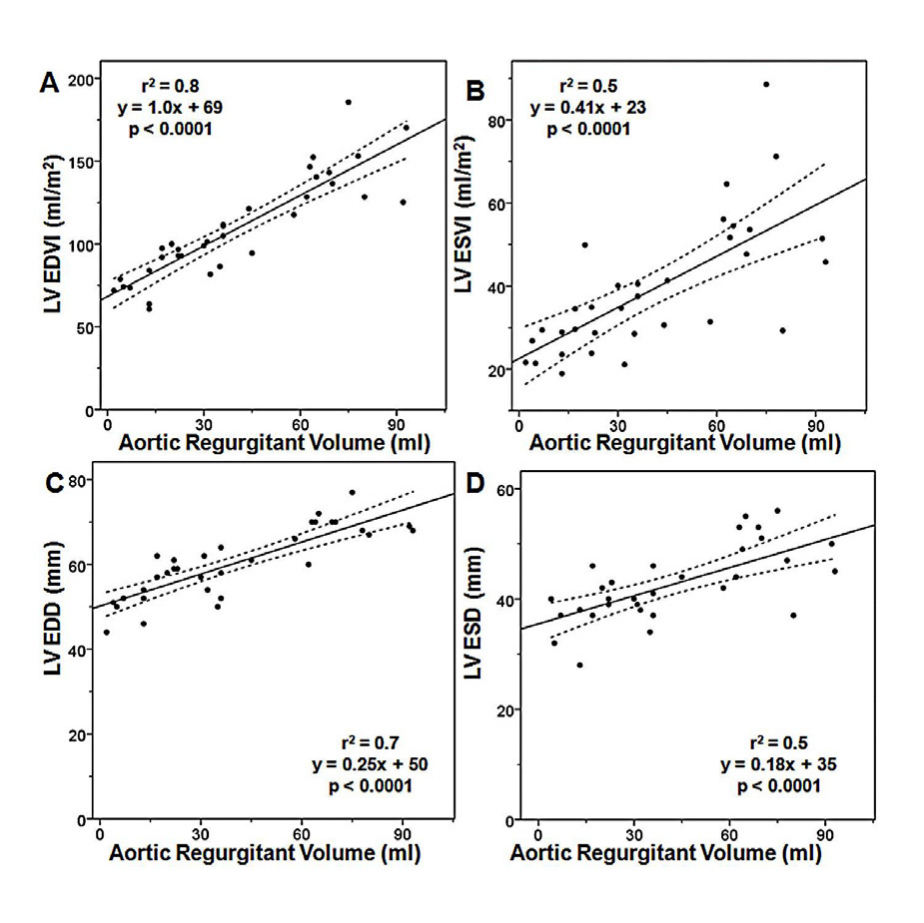
**Relationship Between Aortic Regurgitant Volume and (A) LV EDVI, (B) LV ESVI, (C) LV EDD, and (D) LV ESD**. EDD: end-diastolic dimension, EDVI: end-diastolic volume index, ESD: end-systolic dimension, ESVI: end-systolic volume index, LV: left ventricular.

Demographic and CMR data of the patients with AR are summarized in Table 1. Of the 34 patient studies with AR, 13 (38%) have mild AR, 10 (30%) have moderate AR, and 11 (32%) have severe AR. The mean aortic regurgitant volume is 40 ± 27 ml. The mean LV ejection fraction is 64 ± 7.5%. For patients with mild, moderate or severe AR, the mean LV end-diastolic volume (EDVI) is 83 ± 13 ml/m^2^(range = 61 - 100 ml/m^2^), 103 ± 13 (range = 82 - 121 ml/m^2^), and 146 ± 19 ml/m^2^(range = 125 - 186 ml/m^2^) respectively. Figure 1 shows the relationships between regurgitant volume and LV end-diastolic volume index (EDVI), LV end-systolic volume index (ESVI), LV EDD, and LV ESD. There is a strong, linear relationship between aortic regurgitant volume and LV EDVI (r^2 ^= 0.8). There are moderate, linear relationships between aortic regurgitant volume and LV ESVI (r^2 ^= 0.5), LV EDD (r^2 ^= 0.7), and LV ESD (r^2 ^= 0.5). Linear regression is used to determine the LV volumes that correspond to an LV ESD of 55 mm and an LV EDD of 75 mm. These values are listed in Table 2.

**Table 1:** Clinical and CMR data

	Mitral Regurgitation (n = 23)	Aortic Regurgitation (n = 34)
**Age (years)**	55 ± 15	62 ± 17
**Male sex**	15 (65%)	30 (88%)
**Regurgitant volume (ml)**	46 ± 34	40 ± 27
**LV EF (%)**	72 ± 6.4	64 ± 7.5
**LV ESD (mm)**	37 ± 4.9	42 ± 6.7
**LV EDD (mm)**	58 ± 5.8	60 ± 8.2
**LV EDV (ml)**	178 ± 56	215 ± 67
**LV ESV (ml)**	49 ± 18	76 ± 32
**LV EDVI (ml/m^2^)**	94 ± 23	109 ± 31
**LV ESVI (ml/m^2^)**	26 ± 8.0	39 ± 16
**LV mass index (g/m^2^)**	79 ± 21	96 ± 24
**RV EF (%)**	58 ± 8.4	59 ± 7.3
**RV EDV (ml)**	156 ± 40	156 ± 43
**RV ESV (ml)**	67 ± 30	64 ± 23
**RV EDVI (ml/m^2^)**	80 ± 16	78 ± 15
**RV ESVI (ml/m^2^)**	34 ± 12	33 ± 10

Values are n (%) or median ± standard deviation.

EDD: end-diastolic dimension, EDV: end-diastolic volume, EDVI: end-diastolic volume index, EF: ejection fraction, ESD: end-systolic dimension, ESV: end-systolic volume, ESVI: end-systolic volume index, LV: left ventricular, RV: right ventricle.

**Table 2:** Corresponding LV volumetric parameters to standard linear LV dimensions

	LV Volume Index (ml/m^2^)	LV Volume (ml)
**Mitral Regurgitation**		
**ESD = 40 mm**	ESVI: 29	ESV: 58
		
**Aortic Regurgitation**		
**ESD = 55 mm**	ESVI: 63	ESV: 127
		
**EDD = 75 mm**	EDVI: 158	EDV: 324

EDD: end-diastolic dimension, EDVI: end-diastolic volume index, ESD: end-systolic dimension, ESV: end-systolic volume, ESVI: end-systolic volume index.

### Mitral regurgitation

**Figure 2:**
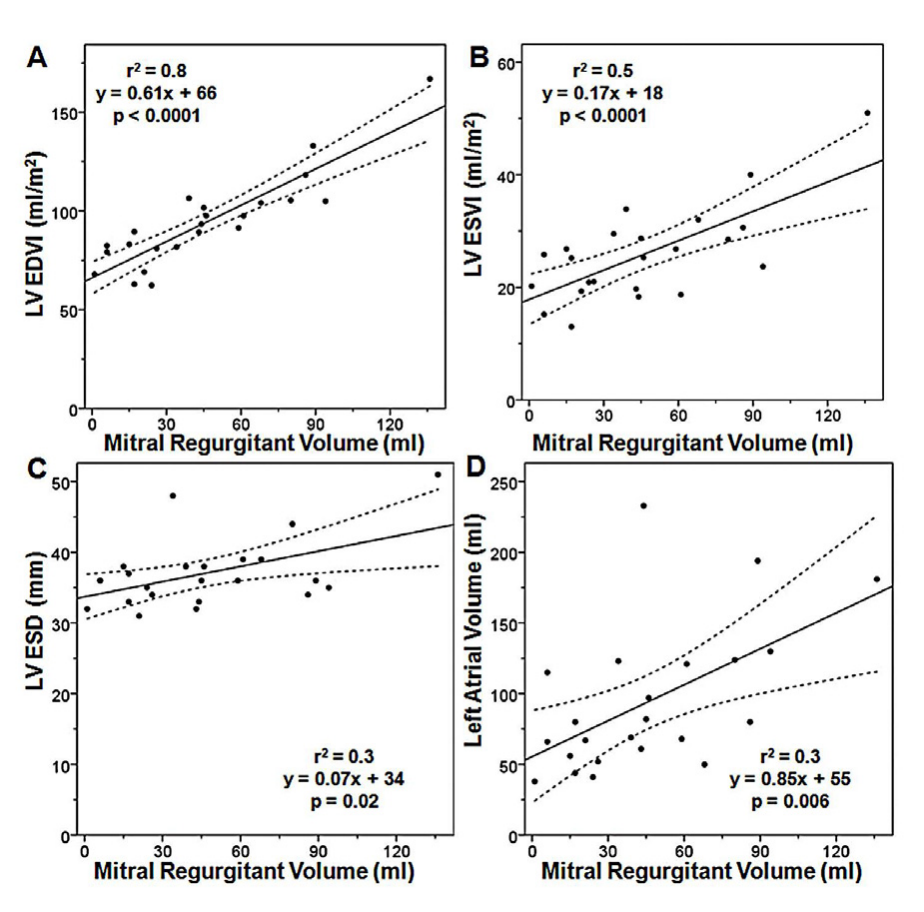
**Relationship Between Mitral Regurgitant Volume and (A) LV EDVI, (B) LV ESVI, (C) LV ESD, and (D) Left atrial volume**. EDD: end-diastolic dimension, EDVI: end-diastolic volume index, ESVI: end-systolic volume index, LV: left ventricular.

Demographic and CMR data of the patients with MR are summarized in Table 1. Of the 23 patient studies with MR, 9 (40%) have mild MR, 7 (30%) have moderate MR, and 7 (30%) have severe MR. The mean mitral regurgitant volume is 46 ± 34 ml. The mean LV ejection fraction is 72 ± 6.4%. For patients with mild, moderate or severe MR the mean LV EDVI is 75 ± 10 ml/m^2^(range = 62 - 89 ml/m^2^), 94 ± 8 ml/m^2^(range = 82 - 107 ml/m^2^) and 119 ± 24 ml/m^2^(range = 98 - 167 ml/m^2^) respectively. Figure 2 shows the relationships between regurgitant volume and LV EDVI, LV ESVI, LV ESD, and LA volume. There is a strong, linear relationship between mitral regurgitant volume and LV EDVI (r^2 ^= 0.8). There is a moderate, linear relationship between mitral regurgitant volume and LV ESVI (r^2 ^= 0.5). There are weak linear relationships between mitral regurgitant volume and LV ESD (r^2 ^= 0.3) and mitral regurgitant volume and LA volume (r^2 ^= 0.3). Linear regression is used to determine the LV volume that corresponds to an LV ESD of 40 mm (shown in Table 2).

### Interobserver agreement

**Figure 3:**
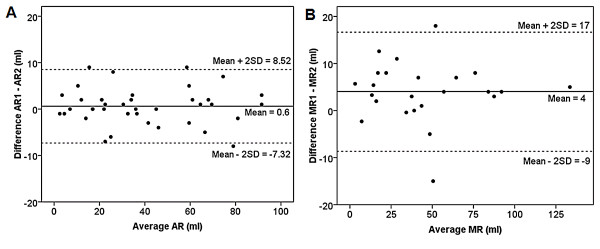
**Bland-Altman analysis of the Interobsever Agreement for the Measure of (A) Aortic and (B) Mitral Regugitant Volume**. AR: aortic regurgitation, MR: mitral regurgitation.

Figure 3 shows strong interobserver agreement in the quantification of AR. The mean ± SD for aortic regurgitant volume was 39 ± 26 ml for observer 1 and 40 ± 26 ml for observer 2 with a strong correlation (Intraclass Correlation Coefficient = 0.99 95% CI 0.989 - 0.997, p < 0.0001). For aortic regurgitant volume, Bland-Altman analysis revealed a strong agreement between observer 1 and 2 with a mean difference of 0.6 ± 4 ml (95% CI -8.52 to 7.32 sml). Figure 3 also shows strong interobserver agreement in the quantification of MR. The mean ± SD for mitral regurgitant volume was 47 ± 32 ml for observer 1 and 43 ± 32 ml for observer 2 with a strong correlation (Intraclass Correlation Coefficient = 0.99 95% CI 0.95 - 0.99, p < 0.0001). For mitral regurgitant volume, Bland-Altman analysis revealed a strong agreement between observer 1 and 2 with a mean difference of 4 ± 6 ml (95% CI -9 to 17 ml).

### The relationship of LV EDVI and mass index to the type and severity of regurgitation

**Figure 4:**
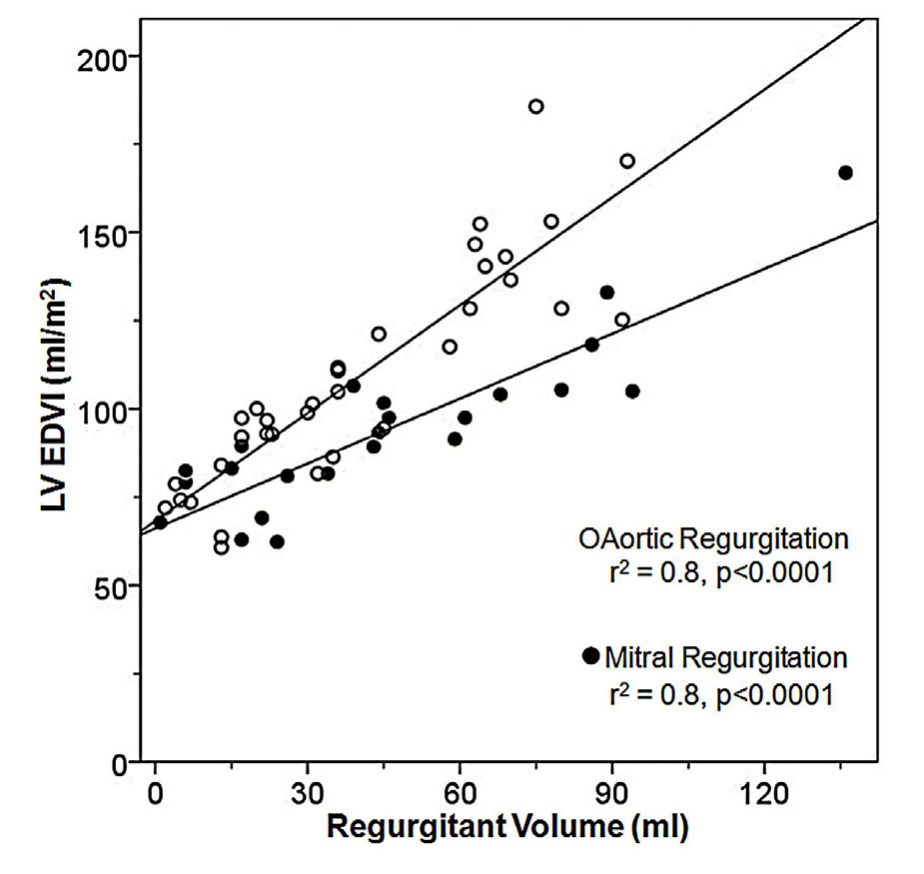
**Relationship Between of Aortic and Mitral Regurgitant Volume and LV EDVI**. EDVI: end-diastolic volume index, LV: left ventricular.

**Figure 5:**
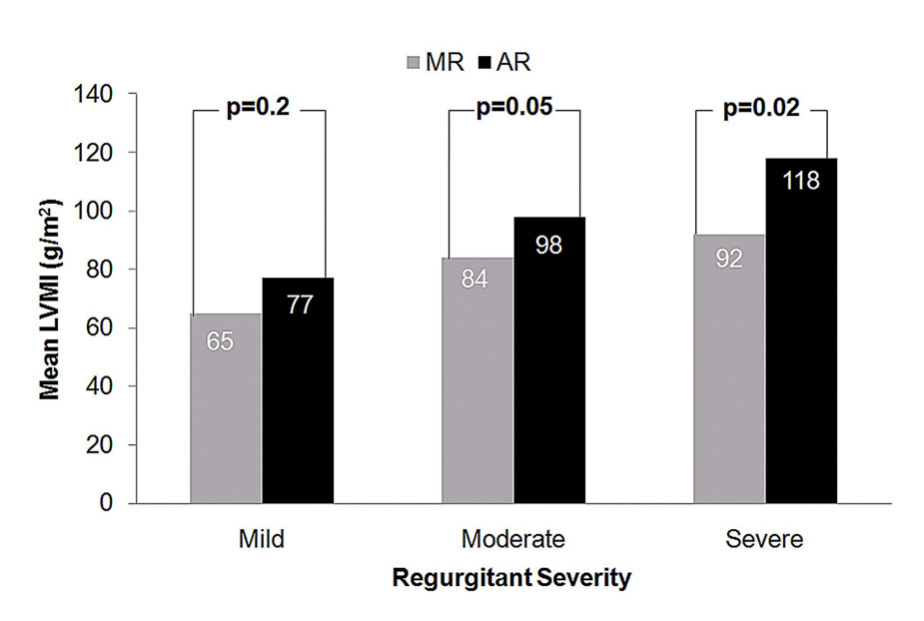
**Comparison of mean LV mass index according the type of regurgitant lesion and the severity of the regurgitant lesion**. AR: aortic regurgitation, LVMI: left ventricular mass index, MR: mitral regurgitation.

We found a greater increase in LV EDVI for a given regurgitant volume in patients with AR compared to those with MR (Figure 4). Furthermore, when comparing mean LV mass index among patients with moderate and severe regurgitation, those with AR had significantly higher LV mass index than those with MR (Figure 5). Mean LV mass index was significantly higher in patients with severe AR vs. mild AR (118 ± 22 vs. 77 ± 17 g/m^2^, p < 0.0001) and severe MR vs. mild MR (92 ± 16 vs. 65 ± 20 g/m^2^, p = 0.01).

## Discussion

CMR has long been accepted as an accurate and reproducible technique for evaluating LV size and function. Good correlation has been obtained by comparing CMR to other established techniques such as echocardiography [11,16], contrast ventriculography [17], and radionuclide imaging [18,19]. In addition, several groups of investigators have documented the accuracy of CMR for quantifying regurgitant volumes in patients with AR or MR, again by comparison to other imaging modalities such as echocardiography [6,20-22] or cardiac catheterization [5]. A problem with these comparative studies is that it can be difficult to determine which of the two imaging modalities is correct when the results differ, which they inevitably do to at least some extent.

To our knowledge, this is the first CMR study to quantify the physiologic relationship between regurgitant volume and chamber volume in patients with isolated chronic AR or MR and preserved LV function. For both AR and MR, we find a strong linear correlation between regurgitant volume and LV EDVI (r^2 ^= 0.8). This provides an independent line of evidence supporting the accuracy of MRI for quantifying regurgitant volume and ventricular volumes. We also find MRI is a robust technique. The data show excellent interobserver variation for quantifying regurgitant volume as demonstrated by the Bland-Altman analysis which showed a mean difference of 0.6 ± 4 ml for AR and 4 ± 6 ml for MR.

In our patients with chronic mitral regurgitation, we find the correlation between regurgitant volume and LV EDVI is stronger than with LV ESVI, probably in part because additional factors influence LV ESVI, such as myocardial contractility. The coupling between LV EDVI and regurgitant volume is strong. An r^2 ^of 0.8 implies 80% of the observed variation in LV volume is due to the severity of the regurgitant volume. This implies that in our patient population only 20% of the variation is related to other factors such as differences in pre-load, afterload or heart rate. It is possible that an acute physiologic change could weaken the coupling between regurgitant volume and LV EDVI, because regurgitant volume is an instaneous measure whereas LV EDVI is a more chronic measure, and may better reflect the long term average severity of regurgitation. It is interesting to note that LV ESD, a parameter included in the AHA/ACC management guidelines to guide therapy, correlates very poorly with regurgitant volume (r^2 ^= 0.2). Finally, left atrial volume, a parameter which is often used by the echocardiographic community and which has been reported as a reliable estimator of regurgitant volume [23], shows a relatively poor correlation with regurgitant volume (r^2 ^= 0.3).

In our patients with chronic AR, we find a stronger positive correlation with LV EDVI than with LV ESVI, again probably in part because additional factors influence LV ESVI, such as myocardial contractility. It is interesting to note that for a given regurgitant volume, patients with AR have more LV enlargement and a greater LV mass index than patients with MR. This observation is likely due to the nature of the hemodynamic stress placed on the LV; whereas MR is a pure volume lesion AR is both a pressure and a volume lesion [24]. Additionally, our data suggest that the difference between the two regression lines reflects the difference in afterload of the two patient groups. As one expects, the two linear regression lines approach the same value at a regurgitant volume of 0. Commonly used linear measures such as LV EDD and LV ESD showed a substantially worse correlation with regurgitant volume than volumetric measures such as LV EDVI or LV ESVI. These finding are consistent with previous reports that a linear measurement does not always accurately represent the actual volumetric changes of a 3-dimensional structure such as the LV [25].

Many of the patients in this study were referred for CMR because the severity of the regurgitation could not be accurately determined by echocardiography. The clinical CMR reports included the regurgitant volume, LV dimensions, and LV volumes. Unfortunately, the LV volumes could not be used by the referring physician to determine the timing of surgical intervention because the AHA/ACC management guidelines only refer to linear dimensions. However, the fact that the volumetric measures correlated better to the regurgitant volume than corresponding linear measures suggests the possibility that volumetric measures may be more valuable than linear measures in assessing the response of the left ventricle to chronic MR or AR and in guiding the timing of surgical intervention. It is our hope that the volumetric correlates to the guideline dimensions which are listed in Table 2 will provide a foundation for the inclusion of volumetric data in future management guidelines.

## Study limitations

Our data are retrospective in nature and only apply to patients with normal or mildly decreased left ventricular function and no other known cardiac disease. These data may not be applicable to patients with other concurrent cardiac diseases and/or LV dysfunction.

## Conclusion

CMR is a robust technique for quantification of regurgitant volume in patients with AR or MR and preserved LV function. LV volumes show a stronger correlation with regurgitant volume than the linear dimensions recommended in the current management guidelines. These data suggest that LV volumes better reflect ventricular remodeling in patients with isolated mitral or aortic regurgitation.

## Abbreviations

AR: aortic regurgitation; CMR: cardiac magnetic resonance; EDD: end-diastolic dimension; EDVI: end-diastolic volume index; ESD: end-systolic dimension; ESVI: end-systolic dimension index; LV: left ventricular; MR: mitral regurgitation; MRI: magnetic resonance imaging; RV: right ventricular.

## Competing interests

The authors have no competing interests that relate to this study. Dr Steven Wolff is the owner of NeoSoft, LLC and NeoCoil, LLC.

## Authors' contributions

SU and SDW designed the study, performed data analysis, and prepared the manuscript. AS performed statistical analysis. OS and CC performed image acquisition. SK, PN performed data collection. FC performed data analysis. All authors read and approved the final manuscript.
